# How are people with obesity managed in primary care? – results of a qualitative, exploratory study in Germany 2022

**DOI:** 10.1186/s13690-023-01214-z

**Published:** 2023-11-13

**Authors:** Julian Wangler, Michael Jansky

**Affiliations:** University Medical Center of the Johannes Gutenberg University Mainz, Centre for General Medicine and Geriatrics – Mainz, Mainz, Germany

**Keywords:** Obesity, Overweight, General practitioner, Therapy, Management

## Abstract

**Background:**

Counselling and management of overweight and obesity are tasks for which general practitioners possess favourable qualifications. Based on a long-term doctor-patient relationship, GPs have various options for actions to deal with overweight problems. To date, however, there is only little evidence on the experiences which people with obesity have made with the primary physician model and the care needs and wishes they actually address to their GPs. This study investigated what experiences people with obesity have had with GP care and what care needs and wishes they communicated to their GPs. The results will be used to derive starting points for optimising the primary healthcare setting.

**Methods:**

A total of 32 individuals affected by obesity were recruited over 24 online health forums. With them, we conducted qualitative interviews in the time between April and October 2022.

**Results:**

The respondents considered the primary care physician to be the central contact person when they sought advice and support with weight problems. The advice of the GP was associated with an increased willingness to deal with reducing one’s own weight. Despite this positive perception, widespread drawbacks existed from the perspective of the respondents: (1) incidental or late discovery of obesity, (2) absence of continuous weight counselling, (3) no agreement on specific weight reduction goals, (4) no referrals to help and support services, (5) insensitive discussion. Only some of the respondents who have recently been able to reduce their weight sustainably attributed their success primarily to the support they received from their GP.

**Conclusion:**

GPs should be encouraged to address obesity consistently and promptly. In addition, concrete recommendations and realistic goals for weight loss should be formulated. Continuous and motivating discussions are crucial in this regard. A focus on nutrition and exercise counselling in the GP’s office should also be encouraged. GPs should be strengthened in their role as mediators by integrating their patients into a network of further assistance as needed. The development of care structures for obesity management should be promoted.

**Supplementary Information:**

The online version contains supplementary material available at 10.1186/s13690-023-01214-z.

**Table:** 

**Text box 1. Contributions to the literature**
• Beyond individual international findings, there is a lack of current studies for German-speaking countries that focus on overweight and obesity management by GPs. In particular, the experiences and care needs of the patients have hardly been examined.
• Despite the favourable conditions of the primary care setting, the results provide evidence that the potential of primary care for overweight and obesity management is currently not being fully exploited.
• The study has made a contribution to recognized gaps in the literature by revealing conditions and predictors of the effectiveness of generalpractice support with regard to the management of people with obesity.

## Background

In recent years, obesity has received increased attention as a chronic disease [1, 2]. Current figures for Germany indicate that 53% of adults are overweight (BMI 25-29.9 kg/m²) and of these 17% are obese (BMI > 30 kg/m²) [1–3]. For Europe, obesity is thought to be significantly involved in the development of approximately 80% of incident cases of type 2 diabetes mellitus, approximately 35% of all ischaemic heart diseases, and approximately 55% of all hypertensive diseases [4]. In addition, the clinical picture is associated with considerable mental distress [5–7]. A large meta-study of evidence-based international guidelines for the treatment of people with weight problems revealed that most guidelines come to the conclusion that overweight and obesity should be treated in a multidisciplinary manner like a chronic disease [8]. In this context, BMI should be included as a routine indicator. A multifactorial, comprehensive lifestyle programme that comprises reduced caloric intake, increased physical activity, and measures supporting a behavioural change for at least 6 to 12 months is recommended [9]. After weight loss, long-term weight maintenance interventions will be required. In summary, there was considerable agreement in the evidence-based international guidelines that overweight and obesity management should be implemented as an elementary constituent of medical healthcare.

When it comes to counselling and managing people with obesity, primary care is particularly significant [10]. GPs often have a long history of familiarity with their patients, hence it is likely that the good relationship of personal trust between physician and patient might have a positive impact on treatment when dealing with a sensitive issue such as obesity [11, 12].

Apart from counselling and support, GPs have several options at their disposal that serve the purpose of effecting weight reduction based on lifestyle changes [13]. This may include dietary and exercise therapy counselling, therapeutic interventions, or a referral to external help services. Patients in need of special psychosocial stabilisation might benefit from psychological intervention. Medication and surgical therapy options may also be taken into consideration [1, 2].

Previous international studies indicated that GPs are aware of the importance of primary care in overweight and obesity management [14]. However, they often have negative attitudes towards people with obesity (e.g., insinuation of lacking willpower) [15–17]. Self-report research suggests that many GPs hold severely biassed and incongruent views about the aetiology of obesity, about people with overweight and obesity [18–20]. In a representative survey of over 600 GPs in southeastern France, Bocquier et al. have shown that approximately a third of respondents had (very) negative attitudes towards people with obesity; 57% were pessimistic about patients’ ability to lose weight [14]. Schwenke et al. were able to identify stigmatising attitudes towards patients with obesity in a clinical study with 47 GPs using the Fat Phobia Scale (FPS) [21]. Younger GP age, male gender and a lower number of referrals to specialists were associated with higher levels of stigmatising attitudes. A qualitative study by Teixeira et al. was also able to show that GPs tend to regard people with obesity as being unmotivated and non-compliant [22]. Correspondingly, a lack of belief in the efficacy of nutrition or exercise therapies was found among a subset of GPs. As a result of subliminal stereotyping, insensitive and inconsistent communication on behalf of the physician might occur in cases of obesity [11]. Often, GPs seem to prefer assuming a more passive attitude and regard the patient to be primarily responsible for his (or her) weight loss [23, 24]. Along with this, treatment plans for weight loss are highly individualised [25]. A lack of adequate structures and programmes is discussed as another reason for the reservation of GPs in obesity management [26–28].

The results of previous studies vary widely with regard to the willingness of people with obesity to seek advice and, if necessary, therapeutic support from their GPs. Surveys conducted in Australian and Israeli practices, for example, showed that patients who attributed an important mediating role to their GPs in terms of weight management, welcomed regular GP advice on dietary issues and physical activity, and showed a high willingness to accept lifestyle changes [28, 29, 30]. Several papers stated that overweight individuals who had received advice regarding their weight from their GPs often made greater efforts to lose weight [13, 31].

On the other hand, surveys in the USA showed that patients had little interest in counselling services and that the physicians’ attempts to initiate lifestyle changes were often not accepted [32]. Patients often did not raise the issue of obesity on their own initiative during consultations, but expected appropriate advice from their attending physician [33]. Other studies found that people with obesity were often dissatisfied with the support they received from their GP. In many cases, the diagnosis of overweight and obesity was not accompanied by specific advice or instructions on diets or physical activity [34, 35].

Beyond individual international findings, there is a lack of current studies for German-speaking countries that focus on overweight and obesity management by GPs. In particular, the experiences and care needs of the patients have hardly been examined. The present study investigates how people with obesity experience the support provided by GPs and what their needs and wishes with regard to obesity management are. The results will be used to determine starting points for an optimisation of primary care.

## Methods

### Guidelines and recruitment

In order to address the research interest, we developed a semi-structured interview guide. It was based both on a relevant literature search and a preliminary study [36]. The topics were the importance of the GP in obesity prevention, the content and form of the GP’s consultation, the effect and assessment of the consultation, potential therapies, general care needs, and possibilities for improving the GP’s care.

The respondents were recruited on 24 online health forums focusing on obesity as their main topic. For this purpose, a call was launched to provide information about the general topic. Individuals who were willing to partake in an interview (no incentives) could contact us via a specified email address.

Subsequent to the voluntary recall of the interested persons (a total of 42 persons from eight of the twelve forums), height, weight, age, gender and living environment (based on the number of inhabitants; general differentiation according to the categories of large city, medium-sized city, small town and rural community) were collected in advance. The criterion to be fulfilled for inclusion was that the individuals to be recruited had been diagnosed with significant overweight (defined as BMI > 25 kg/m²) by a physician or other healthcare professional during the last two years.

In summary, we selected 32 subjects with the highest reported body weight from the abovementioned pool of 42 interested test subjects and recruited them for the study. Choosing the subjects with the highest reported weight was a pragmatic decision, as we felt the likelihood of obtaining valuable information on the topic at hand was greatest. Correspondingly, we had a preference for obese and severely overweight patients according to the focus of the study. The limitation to 32 interviews was, first, due to the nature of the qualitative-exploratory study; secondly, the resources in the project were limited; thirdly, the pool of available subjects was also comparatively small.

### Procedure and sample

All interviews were conducted in rotation by the authors in the time between April and October 2022. In addition to one telephone interview, the option of conducting an interview via chat was also offered. The idea was that, overweight and obesity being a socially sensitive topic, respondents would be more willing to provide truthful information about their situation and experiences with medical care when guarded by the greater anonymity provided by a chat interview. Ultimately, 20 individuals chose in favour of a telephone interview, whereas in 12 cases interviews were conducted via chat. The interviews lasted between 35 and 65 min.

In advance, the respondents were informed about the topic of the interview and received an informed consent form. The latter included the assurance of strict pseudonymisation as well as deletion of the conversation recordings or chat protocols after completion of the evaluation.

Table 1 shows the sample obtained:

**Table 1:** Sociodemographic description of the sample, recruited in the course of the qualitative, exploratory study in Germany 2022 (N = 32)

Age	mean 49 years (median 50 years)
Sex	20 female, 12 male
BMI	mean 28 kg/m², of which 14 > 30 kg/m²; range: 8.7
Living environment	16 metropolitan, 16 small town /rural

### Ethics approval and consent to participate

During this study, no patient data was gathered or clinical tests performed. All 32 individual interviews were strictly pseudonymised. The Ethics Commission of the State of Rhineland-Palatinate, Germany, informed us that approval by an ethics committee was not necessary for a study that does not involve patient data.

Written informed consent for participation and the recording was obtained from all participants before the start of the study.

### Data analysis

The transcripts created after data collection were evaluated in teamwork by applying a qualitative content analysis according to Mayring [37] (using MAXQDA software). First, we elaborated the meaningful basic statements, then we further abstracted and summarised them until a system of categories was finally created which was closely oriented to the guideline. It was repeatedly checked and, if necessary, modified as the evaluation progressed. Our focus was set on a logical categorisation of the different experiences, perspectives and needs. COREQ was used as the reporting statement [38].

The category system we created is divided into five main categories (subcategories in brackets):

1. Role of the GP in the care and treatment of patients with obesity problems (a) competences of the GP; b) readiness to accept and implement the recommendations given by the GP).
2. Experiences with care provided by the GP (a) reason for weight counselling; b) initiator of weight counselling; c) interval or regularity of weight counselling)
3. Subjects of weight counselling: (a) identification of causes; b) information about overweight consequences; c) agreement on weight-loss goals/strategies; d) therapy plan and success criteria; e) dietary counselling; f) exercise counselling; g) other services or assistance)
4. Satisfaction with care provided by the GP (a) assessment of behaviour and communication; b) assessment of time commitment; c) consideration of the patient’s own ideas; d) assessment of the weight loss concept; e) physician responses and goal adjustment; f) implications for the patient’s own motivation; g) implementation and success of weight loss)
5. Articulation of care needs (a) focus and nature of primary care physician support; b) starting points for improvement; c) promotion of physician-patient relationship and motivation).

Theoretical saturation became apparent after 24 interviews. Regardless, we had set the condition that all arranged 32 interviews should be carried out.

## Results

### Role of general practitioners in the care and treatment of obesity

In principle, all respondents considered the GP to be the right contact person to advise patients in matters of prevention of overweight and related risk factors, and the one to take therapeutic measures if necessary. Many respondents emphasised that the GP is in the best position to “pick up patients at their own situation” (I-2 m) and to support them in their overweight management on the basis of an established relationship of trust in the long term.

*“I believe that the general practitioner is the best possible authority to turn to, for only he/she knows me well enough and is aware of the problems I have. He/she is who I trust most.” (I-10f)*.

In the opinion of most respondents (24), the advice and assistance given by their GPs was of greater importance than the recommendations of other physicians or health professionals when it came to achieving weight loss and promoting a healthy lifestyle.

### Experiences with general practitioners

**Fig. 1:**
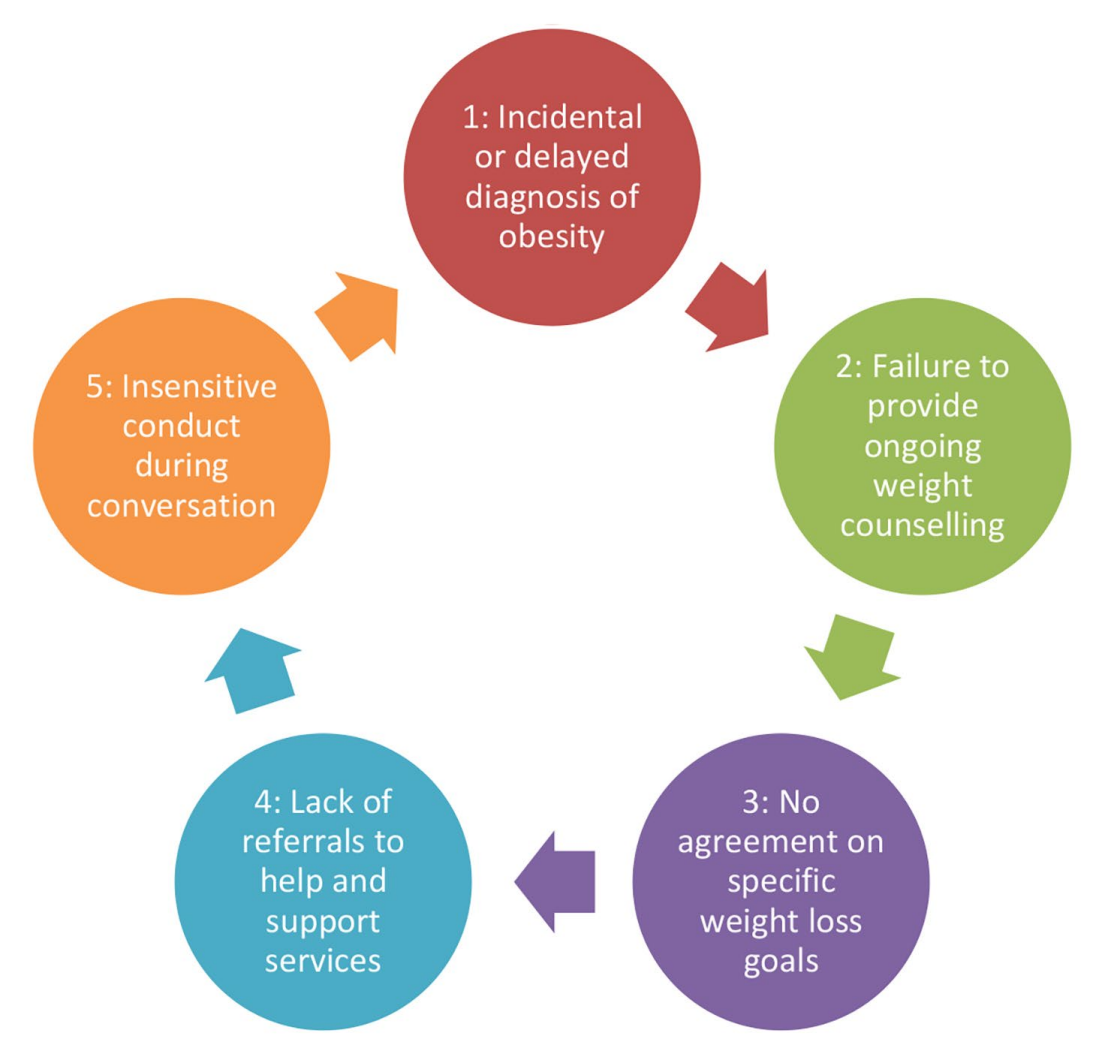
Identified problem areas of obesity care provided by general practitioners

In the course of the interviews, we identified several problem areas of GP care provision of people with obesity (see Fig. 1).

30 of the 32 respondents stated that they had already discussed their weight with their current or a previous GP. In some cases, check-up examinations were mentioned as occasions for weight counselling, participation in a disease management programme or a treatment of diabetes were also mentioned in individual cases. The majority, however, stated that there had been no specific reason and that weight had been discussed rather casually (see Fig. 1, point 1).

*“The fact that we came to talk about my overweight was more or less coincidental. It was not actively controlled now.“ (I-18f)*.

18 respondents stated that the doctor originally had taken the initiative and brought up the subject of obesity; whereas 12 other persons had consulted the GP.

*“I have been consulting my general practitioner for a long time, but he has never approached me openly about the subject. […] We just stumbled upon it at some point.“ (I-22f)*.

According to statements made by 12 persons, weight counselling took place once, for 6 persons more often than once, and 12 respondents stated that overweight issues had become a recurring topic of conversation since it had been brought up the first time. However, a majority (20) admitted that conversations about the weight situation had taken place earlier or very irregularly and that it often had not been foreseeable for them when the topic would come up again (see Fig. 1, point 2).

### Issues of weight counselling

In almost all cases (28), the GPs emphasised the negative consequences of obesity. After the overweight had been identified, some of the physicians (14) were also concerned to find out possible causes so that individual needs could be better addressed.

*“No, he didn’t ask more specifics on that. He then very quickly moved on to the recommendation.“ (I-18f)*.

Based on the agreement between physician and patient that weight reduction should be aimed for, a majority (22) described that a moderate reduction in weight was recommended with the goal of maintaining the new weight. However, only 6 individuals reported that specific goals were agreed upon. Most often. the time frame in which progress should be made also remained an open question (see Fig. 1, point 3).

Twenty-four respondents stated that general dietary counselling had been provided. The physicians frequently had recommended a low-calorie diet and given specific advice such as avoiding certain foods or substituting certain products (“lots of vegetables, little meat”). Other patients had been advised to reduce the amount of food they ate and change their eating rhythm (“regular eating habits”), or even their ‘eating culture’ (“conscious eating”). In individual cases (6), the GP would hand out a diet plan. In two cases, an app specifically for dietary change had been recommended. Beyond the actual consultation and isolated measures such as diet plans, the physicians rather rarely recommended additional help and support services. 6 persons mentioned referrals to dietary counselling, health insurance offers, self-help groups, special cooking courses or spa stays.

While nutrition was addressed comparatively often, only a smaller number of respondents recalled exercise counselling. Beyond general references to the importance of regular exercise, 10 respondents described suggestions for physical activities (e.g., getting an exercise bike, joining a cardiac sports group, swimming, walking). Hardly any concrete suggestions were made concerning the frequency and intensity of physical activities. 4 persons stated that the GP had referred them to concrete offers of help (e.g., health fitness centres, courses offered by community colleges).

### Satisfaction with general practitioner care

Most respondents (18) positively rated that the GP had principally signalled their willingness to help, had pointed out the risk factors of being too overweight and had responded to queries. In addition, a partnership-like relationship was praised (16).

*“The trust level is just very high, yes. So the preconditions are already fulfilled.“ (I-6 m)*.

However, many respondents criticised the care which their GPs actually provided. In particular, they strongly expressed that no continuous guidance had taken place; often one or two relatively short conversations on the subject of overweight was all they had (see Fig. 1, point 2).

“*It was kind of like this: ‘Once and then never again.‘ It just didn’t amount to much that way. There was nothing like continuous care worth mentioning.“ (I-14 m)*.

Another widespread point of criticism, also voiced by those respondents for whom the GP had suggested specific measures for weight reduction, had to do with the absence of success criteria. Since there had been no goals to be achieved beyond general recommendations, they had lacked an orientation benchmark and a motivating element (see Fig. 1, point 3).

“*It would have helped me a lot if I had known when to achieve what. I mean goals that are set specifically for you.“ (I-24 m)*.

*“In what time should I achieve what and how? These questions have not been clarified exactly. And if you lack that, then you don’t have a compass when you lose weight. […] Then the whole thing is quickly doomed to failure.“ (I-28f)*.

According to their own statements, ten respondents had been successful in reducing their weight noticeably and sustainably in recent years or months. This is attributed to the support of the GP by 4 persons. A huge number of the respondents (18) refer to what they see as a lack of therapeutic support. Apart from general counselling, there had been a “lack of a coherent, clear concept [of] how the pounds should fall off” (I-2 m).

*“You can’t call it therapy. Just a ‘you should do this and could do that’” (I-30m).*

In addition, from the point of view of a majority of the respondents (20), the GPs made too few complementary offers in the process of care as to what help and support options were available for continuous weight loss and an increase in fitness in the local vicinity (see Fig. 1, point 4).

*“No, there was far too little coming. It seemed to me that he didn’t know much about such courses either, like what kind of possibilities existed.“ (I-18f)*.

*“I felt a bit fobbed off. ‘Take some kind of course’. Which one? What? Where is there something here?“ (I-30 m)*.

Ten respondents mentioned that they felt that there had occasionally been a lack of empathy on the part of GPs when dealing with the weight situation (see Fig. 1, point 5). Situations were described in which rude, arrogant or insulting behaviour on the part of the physicians became apparent. In two cases, this stigmatising behaviour resulted in the termination of the physician-patient relationship.

*“I felt that he was cracking little jokes on me, saying something like: everything will soon collapse under you should you go on like this.“ (I-20f)*.

In addition, a widespread passivity of physicians has been described (14), which had led to patients feeling left alone with their weight problem. This is partly associated with negative attitudes of practitioners towards people with obesity (6).

*“Patients like us have experienced this before […]: Physicians who do not give us much credit. The fat ones can’t get it together, they just can’t stop eating.“ (I-14 m)*.

### Care needs

Regardless of the care actually experienced, the majority of the respondents favoured a proactive approach by GPs when dealing with overweight patients. An open but polite and sensitive approach was advocated.

Central to almost all interviewees are continuous discussions that serve to provide ongoing advice and, not least, motivation. The intervals should ideally be a few weeks in order, for example, to be able to analyse reasons for failure in good time and to try out new approaches.

*“If there are no such deadlines, then you quickly lose sight of it and let things slide. You have to keep at it regularly to overcome your weaker self.“ (I-10f)*.

The respondents attached great importance to agreeing on tangible goals (weight to be reduced, time periods) and measures. In doing so, action steps should be chosen to accommodate patients’ sensitivities and interests.

*“I would like to enrol in a structured diet or exercise programme, teaching me how to lose weight slowly but surely. And also goals that I can measure myself against.“ (I- 28f)*.

Respondents articulated a desire for referrals or referrals to help, whether it be health insurance options, fitness classes, or support groups. The GP is seen as a good platform from which to raise awareness of such flanking offers.

*“If my GP recommended such a course to me, I would definitely be much more likely to accept it than if I had heard about it somewhere else.“ (I-30 m)*.

## Discussion

### Main findings and comparison with prior work

The interviews revealed that people with obesity and overweight consider GPs as key contacts in matters of advice, support and therapy. GPs are associated with the potential to appropriately and continuously address the individual needs of appropriate patients. Based on these assumptions, a considerable proportion of respondents signalled an increased willingness to lose weight when the GP provided counselling, motivation, and referrals [13, 30, 31]. Based on the agreement between GP and patient that weight reduction should be aimed for, a majority described that a moderate reduction in weight was recommended with the goal of maintaining the new weight. Overall, there are no significant differences in our sample between key socio-demographic characteristics such as gender, age or living environment.

Despite these fundamentally positive perceptions of the role of GPs, the study results reveal a number of deficiencies. For example, problems of obesity are often identified rather casually or are addressed with a delay. In addition, many patients experience it as problematic that there is hardly any regular exchange about their own weight situation and that GPs often limit themselves to giving a quite generalised advice on diet and/or exercise. In many cases, the fact that no specific weight loss goals have been agreed upon was perceived as a considerable deficit affecting the patient’s own orientation and motivation. In addition, people with obesity were referred to existing support services in a rather situation-dependent manner. In individual cases, the physician-patient relationship was encumbered by the insensitive behaviour of some physicians, which induced the patients concerned to assume that their physicians doubted that their patients could muster the necessary discipline. Of those respondents who stated that they had recently been able to reduce their weight sustainably, only a small number attributed this success mainly to the support they had received from their GP.

The results fit into the body of evidence that obesity is a polarising condition among physicians and varying degrees of willingness to provide care can be encountered [6, 10, 11, 27, 28]. Previous studies have indicated that GPs often shy away from assuming an active role in obesity management. Reasons for this are seen to exist in a lack of time and resources, but a lack of patient motivation is also assumed [25, 36]. Also of importance is that many GPs feel left to their own devices in a task as comprehensive and long-term as obesity care. Various studies revealed that GPs complain about a deficit of existing structures, which results in a lack of individualisable therapy concepts in which patients can be continuously supported by the GP in the process of their lifestyle change [15, 26, 32, 34]. In addition, it has been shown that knowledge of locally available help and support services to which referrals can be made is often limited, and only a smaller proportion of physicians make consistent use of them, for example, by referring to fitness and exercise providers or psychotherapists [32, 33].

It is noticeable that these are largely conservative therapeutic approaches that GPs take or recommend.In this context, it could be discussed why main causes related to eating and physical activity were addressed. Obesity is a complex disease, often characterized by comorbidities, that should be treated multidisciplinary [9–11]. Various studies and scoping reviews provide evidence that the focus of GPs on nutrition and exercise therapies may have something to do with the (latent) stigmatisation of people with obesity as mentioned in the introduction [6, 14, 20]. Within the sample, best practice examples can be found for motivational consultation and the effort to engage with patients in their personal situation. Such behavioural treatment strategies can play a central role in long-term treatment outcomes [33, 34]. Nevertheless, the doctors’ prescribing behavior implies that, from their perspective, it is almost exclusively about patients working on themselves, whereas more complex interactions between health and social factors tend to be ignored [17, 21]. In this respect, it would be advisable to sensitize GPs so that they are as open as possible to complex causes of obesity and, accordingly, to multi-perspective solutions.

Recent studies emphasised the great value and indispensability of primary care for weight problems in order to effectively encounter the global spread of overweight and obesity [39]. This was accompanied by a greater inclusion of patient needs, interests, and experiences, which can contribute to greater activation, self-determination, and longer-term weight loss success [40]. It seems particularly important that weight loss goals are realistic and achievable [20].

### Implications

From the results of the present study and in connection with further studies, various implications for future research and interventions can be derived. The goal is to specifically optimise primary care support for people with obesity.

Some GPs tend to see the causes of obesity in the patient’s personality. Therefore, it seems advisable to raise awareness among GPs that obesity can have as complex background involving factors such as life circumstances and pre-existing conditions [2, 3, 5].

GPs should be encouraged in their role as mediators by referring their patients to an extended healthcare network including psychotherapists or dietary assistants [41]. For example, health authorities often provide a useful guide to the local training and consultation services available.

Accordingly, developing structured care programmes for obesity management seems sensible. These programmes should not only aim towards improving patient care but also training for GPs and their practice staff. International model projects may provide guidance and could be adapted to suit the specific situation [41–44].

Ideally, GPs should be placed in a position where they can fulfil two main tasks – individual consultation and treatment as well as coordination within a multidisciplinary obesity care network [41, 45].

### Strengths and weaknesses

A qualitative survey has several limitations that should be taken into consideration.

This study was based on self-reported height and weight (BMI), so that incorrect information cannot be ruled out.

The investigation carried out was a non-representative study with a small sample that is not representative of the entirety of primary care patients with a severe degree of obesity. Accordingly, the weakness of this study is the small sample of the population surveyed and with a low degree of obesity since the majority of the population surveyed is overweight.

The recruitment via online forums made it likely that a specific group with needs of specific information and discussion had been recruited for the sample. According to the authors’ impression, the ultimately recruited group showed an overall high interest in being systematically attended and medically advised by their GPs. At the same time, this type of recruitment was a good way to obtain patients who were willing to talk as openly as possible about their problems of overweight and obesity care as provided by GPs.

More than one-third of the interviews were not conducted by telephone, but by chat. It seems obvious that contributions in chat communication do not allow for the same possibilities of articulation and interaction as oral interviews do. In this respect, it should be considered that information might have been lost or simplified here.

## Conclusions

Despite the favourable conditions of the primary care setting, the interviews provide evidence that the potential of primary care for overweight and obesity management is currently not being fully exploited. The following starting points for optimisation therefore seem advisable:

- Overweight problems should be addressed systematically, consistently and promptly in primary care. Useful occasions are, for example, medical check-ups.
- The identification of severe overweight or obesity should be linked to concrete recommendations for action and realistic, individually coordinated targets. Here, existing guidelines provide further assistance and orientation [42].
- Focused nutrition and physical activity counselling in primary care practices appears to be a useful contribution to support obesity prevention. Good practice examples and models have already been presented in this regard [46]. Practice staff could be involved in a supportive manner and receive targeted further training, so that GPs can be relieved by delegation.
- Regular, binding discussions and efforts to ‘pick up’ patients from their personal situation (behaviour-oriented treatment strategies) and to motivate them continuously are important prerequisites for the long-term success of obesity management [47].
- It seems advisable to raise awareness among family physicians that obesity can have complex backgrounds, in which not only the individual lifestyle, but also life circumstances, genetic predisposition and pre-existing conditions are effective.
- Family physicians should be encouraged in their role as mediators by integrating people with obesity into further help networks as needed. Almost all statutory health insurers offer prevention programmes; the same applies to health offices, which often have a good overview of courses and counselling offers in the district [27]. Municipal cooperation networks for health promotion could account for great added value in providing GPs with an overview of existing health services and in referring patients in a targeted manner [47, 48].
- The development of structured, GP-based care programmes for obesity management seems to make sense.

### Electronic supplementary material

Below is the link to the electronic supplementary material.

Supplementary Material 1

## Data Availability

Data from this research are not publicly available because participants did not give permission for recordings or transcripts to be released to other researchers.

## Abbreviations

GP: General Practitioner
