# Supplementary material for: How are people with obesity managed in primary care? – results of a qualitative, exploratory study in Germany 2022
Source: Arch Public Health. 2023 Nov 13;81:196. doi: 10.1186/s13690-023-01214-z (PMC10641940; doi:10.1186/s13690-023-01214-z)
Supplement: Supplementary file 1 — Supplementary Material 1 [file 13690_2023_1214_MOESM1_ESM.docx]

**Aims and Scope statement**

1. What is known?

Previous international studies indicated that GPs are aware of the importance of primary care in overweight and obesity management. However, they often have negative attitudes toward severely overweight patients. Correspondingly, a lack of belief in the efficacy of nutrition or exercise therapies was found among a subset of GPs. As a result of subliminal stereotyping, insensitive and inconsistent communication on behalf of the physician might occur in cases of obesity. Often, GPs seem to prefer assuming a more passive attitude and regard the patient to be primarily responsible for his (or her) weight loss. Along with this, treatment plans for weight loss are highly individualised. A lack of adequate structures and programmes is discussed as another reason for the reservation of GPs in obesity management.

The results of previous studies vary widely with regard to the willingness of overweight and obese patients to seek advice and, if necessary, therapeutic support from their GPs. Surveys conducted in Australian and Israeli practices, for example, showed that patients who attributed an important mediating role to their GPs in relation to weight management, welcomed regular GP advice on dietary issues and physical activity, and showed a high willingness to accept lifestyle changes. Several papers stated that overweight individuals who had received advice regarding their weight from their GPs often made greater efforts to lose weight.

Surveys in the USA on the other hand showed that patients had little interest in counselling services and that the physicians' attempts to initiate lifestyle changes were often not accepted. Patients often did not raise the issue of obesity on their own initiative during consultations, but expected appropriate advice from their attending physician. Other studies found that obese patients were often dissatisfied with the care they received from their GPs. In many cases, the diagnosis of overweight and obesity was not accompanied by specific advice or instructions on diets or physical activity.

2. What does the study add?

Beyond individual international findings, there is a lack of current studies for German-speaking countries that focus on overweight and obesity management by GPs. In particular, the experiences and care needs of the patients have hardly been examined. The present study investigates how overweight and obese patients experience the care provided by GPs and what their needs and wishes with regard to obesity management are. The results will be used to determine starting points for an optimisation of primary care.

1. What are implications for clinical practice, public health and / or research?

Despite the favourable conditions of the primary care setting, the interviews provide evidence that the potential of primary care for overweight and obesity management is currently not being fully exploited. The following starting points for optimisation therefore were derived from the data analysis:

- Overweight problems should be addressed systematically, consistently and promptly in primary care. Useful occasions are, for example, medical check-ups.
- The identification of severe overweight or obesity should be linked to concrete recommendations for action and realistic, individually coordinated targets. Here, existing guidelines provide further assistance and orientation.
- Focused nutrition and physical activity counselling in primary care practices appears to be a useful contribution to support obesity prevention. Good practice examples and models have already been presented in this regard. Practice staff could be involved in a supportive manner and receive targeted further training, so that GPs can be relieved by delegation.
  - Regular, binding discussions and efforts to 'pick up' patients from their personal situation (behaviour-oriented treatment strategies) and to motivate them continuously are important prerequisites for the long-term success of obesity management.
- It seems advisable to raise awareness among family physicians that obesity can have complex backgrounds, in which not only the individual lifestyle, but also life circumstances, genetic predisposition and pre-existing conditions are effective.
- Family physicians should be encouraged in their role as mediators by integrating obese patients into further help networks as needed. Almost all statutory health insurers offer prevention programmes; the same applies to health offices, which often have a good overview of courses and counselling offers in the district. Municipal cooperation networks for health promotion could account for great added value in providing GPs with an overview of existing health services and in referring patients in a targeted manner.
  - The development of structured, GP-based care programmes for obesity management seems to make sense.
